# Deep sequencing discovery of novel and conserved microRNAs in trifoliate orange (*Citrus trifoliata*)

**DOI:** 10.1186/1471-2164-11-431

**Published:** 2010-07-13

**Authors:** Changnian Song, Chen Wang, Changqing Zhang, Nicholas Kibet Korir, Huaping Yu, Zhengqiang Ma, Jinggui Fang

**Affiliations:** 1 College of Horticulture, Nanjing Agricultural University, Nanjing 210095, China; 2 Department of Horticulture, Nanjing Jinling Institute of Technology, Nanjing 210038, China; 3 State Key Laboratory of Crop Genetics and Germplasm Enhancement, Nanjing Agricultural University, Nanjing 210095, China

## Abstract

**Background:**

MicroRNAs (miRNAs) play a critical role in post-transcriptional gene regulation and have been shown to control many genes involved in various biological and metabolic processes. There have been extensive studies to discover miRNAs and analyze their functions in model plant species, such as *Arabidopsis *and rice. Deep sequencing technologies have facilitated identification of species-specific or lowly expressed as well as conserved or highly expressed miRNAs in plants.

**Results:**

In this research, we used Solexa sequencing to discover new microRNAs in trifoliate orange (*Citrus trifoliata*) which is an important rootstock of citrus. A total of 13,106,753 reads representing 4,876,395 distinct sequences were obtained from a short RNA library generated from small RNA extracted from *C. trifoliata *flower and fruit tissues. Based on sequence similarity and hairpin structure prediction, we found that 156,639 reads representing 63 sequences from 42 highly conserved miRNA families, have perfect matches to known miRNAs. We also identified 10 novel miRNA candidates whose precursors were all potentially generated from citrus ESTs. In addition, five miRNA* sequences were also sequenced. These sequences had not been earlier described in other plant species and accumulation of the 10 novel miRNAs were confirmed by qRT-PCR analysis. Potential target genes were predicted for most conserved and novel miRNAs. Moreover, four target genes including one encoding IRX12 copper ion binding/oxidoreductase and three genes encoding NB-LRR disease resistance protein have been experimentally verified by detection of the miRNA-mediated mRNA cleavage in *C. trifoliata*.

**Conclusion:**

Deep sequencing of short RNAs from *C. trifoliata *flowers and fruits identified 10 new potential miRNAs and 42 highly conserved miRNA families, indicating that specific miRNAs exist in *C. trifoliata*. These results show that regulatory miRNAs exist in agronomically important trifoliate orange and may play an important role in citrus growth, development, and response to disease.

## Background

MicroRNAs (miRNAs) are 19-23 nucleotide long non-coding RNAs that regulate gene expression at the post-transcriptional level, either by endonucleolytic cleavage or by translational inhibition [1-3]. Increasing evidence indicates that miRNAs play major roles in key aspects of plant development and their response to environmental stresses [4-7]. The fact that a large number of the known miRNAs in the plant kingdom from mosses and ferns to higher flowering plants are evolutionarily conserved has been used as a practical indicator for identification or prediction of miRNAs by homology searches in other species [8,9]. miRNAs predicted by bioinformatics should be validated for their expression by experimental methods. Northern blotting and PCR based amplification of adaptor-ligated cDNA have been used for validation of predicted miRNAs [10]. Northern blotting might not be sensitive enough to detect less-abundant miRNAs and it does not reveal the actual miRNA sequences while PCR-based amplification can be difficult in practice when the actual mature miRNA region is unknown. Recently developed next-generation high throughput sequencing technologies provide a powerful strategy to identify as well as quantify miRNAs. These technologies open up possibilities of exploring sRNA populations in economically important species that lack adequate genome information, such as trifoliate orange (*Citrus trifoliata*).

Increasing evidence shows that the miRNA repertoire of any plant or animal species comprises of a set of conserved ancient miRNAs as well as many recently evolved species-specific miRNAs [11-13]. Since the species-specific miRNAs often accumulate at lower levels than conserved ancient miRNAs, it is sometimes difficult to assess them when derived from traditional sequencing approaches such as the Sanger sequencing method which has been widely used in model plant species with known genome sequences e.g *Arabidopsis*, poplar, and rice [14]. The availability of next generation sequencing technologies provide high throughput tools to make new discoveries of additional species-specific or lowly expressed miRNAs, e.g. in *Arabidopsis *[12,15], rice [16,17], poplar [18,19], *Triticum aestivum *[20,21], *Zea mays *[22,23], *Medicago tuncatula *[24], *Lycopersicon esculentum *[25], *Gossypium hirsutum *[26], and *Taxus chinensis *[27]. With the availability of high throughput sequencing technology, like the 454 Technology, Solexa Platform, and Massively Parallel Sequencing (MPSS), it may be possible to make new discoveries of species specific or lowly expressed miRNAs. However, there still exist some differences between these novel sequencing technologies. It is reported that the longest reads can be obtained using 454 Technology, while the Solexa platform can yield a higher number of reads [24], and is fit for sequencing of shorter reads (up to 35 bp) [25]. Although MPSS can also give a huge number of reads, it gives even shorter reads i.e only 17 bp [28] that are shorter than that of miRNAs. Since the miRNAs sequences are only about 21 nt in length, the Solexa platform seems to be the preferable choice for miRNA discovery.

Citrus is one of the most economically important evergreen fruit crop in the world. *Citrus trifoliata *(representing *Poncirus trifoliata *in the NCBI taxonomy) has consistently been one of the most important rootstock species used in the citrus industry, and has even been used as a model species for citrus molecular biology and genomic studies. The availability of a large number of expressed sequence tags (ESTs) from *C. trifoliata *is also an excellent source of experimental material for elucidation of gene expression and regulation. Although miRNAs have been extensively studied in the past five years, limited systematic study of miRNAs has been performed on the citrus genus and especially *C. trifoliata*. Sequencing of all expressed sRNAs is required for complete identification of conserved miRNAs in *C. trifoliata*.

Identification of several miRNAs from citrus by computational approaches [9,29,30] and the verification of conserved citrus miRNA by homology to *Arabidopsis *have been reported [31]. However, the number of predicted *C. trifoliata *miRNAs still remains quite low. Recent miRNA analysis in *Arabidopsis *and rice using the deep sequencing approach discovered that the encoding loci of non-conserved miRNAs were more than that of conserved miRNAs [12,16]. Therefore, it was necessary to carry out further research on the miRNAs in *C. trifoliata*, and deep sequencing as a method was given preference.

To investigate the role of miRNAs during reproductive growth, high throughput sequencing technology (Illumina) was employed to survey sRNA populations from *C. trifoliata *flower buds, flowers and fruits at different development stages. In this study, a total of 13,106,753 sRNAs representing 4,876,395 unique sRNAs were sequenced. Based on sequence similarity and hairpin structure prediction, we found that 156,639 reads representing 63 sequences from 42 highly conserved miRNA families, have perfect matches to known miRNAs. Our results indicate that a complex and diverse sRNA population exists in *C. trifoliata*. Based on the identified miRNA and some miRNA* sequences, 29 conversed miRNA (miR156, miR160, miR162, miR164, miR165, miR166, miR167, miR168, miR169, miR170, miR171, miR172, miR319, miR390, miR394, miR396, miR398, miR399, miR403, miR408, miR419, miR530, miR835, miR844, miR950, miR1027, miR1044, miR1426, and miR1446) precursors have been identified from the citrus EST library [9,29-31]. Through deep sequencing we also identified ten species-specific miRNAs whose precursors were all potentially generated from 560,271 citrus ESTs, of which five miRNA* sequences were also sequenced. Furthermore, we studied expression patterns of the 10 novel miRNA candidates by qRT-PCR in different tissues of *C. trifoliata*. Potential target genes were predicted for most conserved and novel miRNAs, of which four target genes including one IRX12 encoding copper ion binding/oxidoreductase and three genes encoding NB-LRR disease resistance protein have been experimentally verified by detection of the miRNA-mediated mRNA cleavage in *C. trifoliata*.

## Results

### *C. trifoliata *has a complex sRNA population

To identify miRNAs involved in development of the citrus rootstock, *C. trifoliata*, a separate sRNA library was generated from the earlier mentioned reproductive tissues. The library was sequenced by Solexa (Illumina), yielding a total of 13,106,573 sRNA raw reads with lengths of 18 to 30nt and consisting of 4,876,395 unique sequences (Table 1). After further removal of tRNAs (265,796), rRNAs (1,643,869), snRNAs (67,225), snoRNAs (30, 909), exon RNA (7,136), intron sense (3,947), and repeat region (67,225), a total of 11,091,865 sRNA sequences were obtained. Although some sRNAs were highly abundant and present thousands of times in our dataset, majority of sRNAs were sequenced only a few times. For example, 4,876,395 out of 13, 113, 220 sRNAs were sequenced only once. The results show that (1) the expression of different sRNAs in trifoliate orange varies drastically and (2) survey of sRNA is far from being exhausted in the trifoliate orange. This also suggests that trifoliate orange contains a large and diverse sRNA population. Compared to equivalent studies from other plants (Table 2), the quantity of sRNAs acquired from *C. trifoliata *is at least 14 times more, which might be due to the difference of depth of the sequencing effort, the genome size of the plants, or the development stages of the samples collected in the different sequencing projects.

**Table 1:** SRNAs annotation and length distribution

SRNA	Unique reads	redundant reads
rRNA	76,683	1,643,869
tRNA	12,410	265,796
snRNA	3,246	67,225
exon RNA	3,358	7,136
snoRNA	1,483	30,909
intron sense	1,406	2,473
repeat region	1,386	3,947
Known miRNAs (exact match)	42	156,639
New miRNA candidates With sequenced miRNA*	23	764
Un-annotated	4,776,358	10,934,462
Total	4,876,395	13,113,220

**Table 2:** High-throughput sequencing of sRNAs in some plants

Species	Tissues	Techniques	Redundant reads ** (× 10**^**4**^**)**	Unique reads ** (× 10**^**4**^**)**	Reference
***Arabidopsis***	Inflorescence and seedling	MPSS	221.01	10.48	Science, 2005, 309 (5740): 1567-1569
	Mixture materials of all developmental stages	454-FLX	88.70	34.00	Genes & Development, 2006, 20 (24): 3407-3425
	Wild-type *Arabidopsis *inflorescence	MPSS	72.10	6.75	Genome Research, 2006, 16 (10): 1276-1288
	Inflorescence of *rdr2 *mutant	MPSS	91.59	1.53	
	Wild-type *Arabidopsis *and *dcl1-7*, *dcl2-1 *and *dcl3-1 *mutant	454-FLX	47.04	21.86	PLoS ONE, 2007, 2 (2): e219.
Rice	Inflorescence, shoot and seedling	MPSS	295.39	28.43	Nature Biotechnology, 2007, 25 (4):473-477
	Mixture materials of all developmental stages	454-FLX	9.23	1.28	Nucleic Acids Research, 2007, 35: 829-833
	Wild-type rice seedling	454-FLX	71.42	5.88	BMC Plant Biology, 2008, 8: 25
	Seedling treated with salt stress			4.30	
	Seedling treated with drought			8.10	
Populus	Leaf	454-FLX	4.13	0.60	BMC Genomics, 2007, 8: 481
	Bud		3.56	0.63	
Wheat	Mixture of leaf, root and spike	454-FLX	26.30	25.25	Genome Biology, 2007, 8 (6): R96
Soybean	Wild-type soybean root	454-FLX	15.91		BMC Genomics, 2008, 9: 160
	*japonicum*-inoculated soybean roots		19.49		
Tomato	Fruit Leaf	454-FLX	53.70 18.48	2.25 10.28	Genome Research, 2008, 18(10): 1602-1609
Maize	Wild-type maize spike	Solexa	560.00		Proceedings of the National Academy of Sciences of the USA, 2008, 105 (39): 14958-14963
	*mop1-1 *mutant		720.00		
*C. trifoliata*	flower buds, flowers, fruits	Solexa	1310.7	487.6	

**Figure 1:**
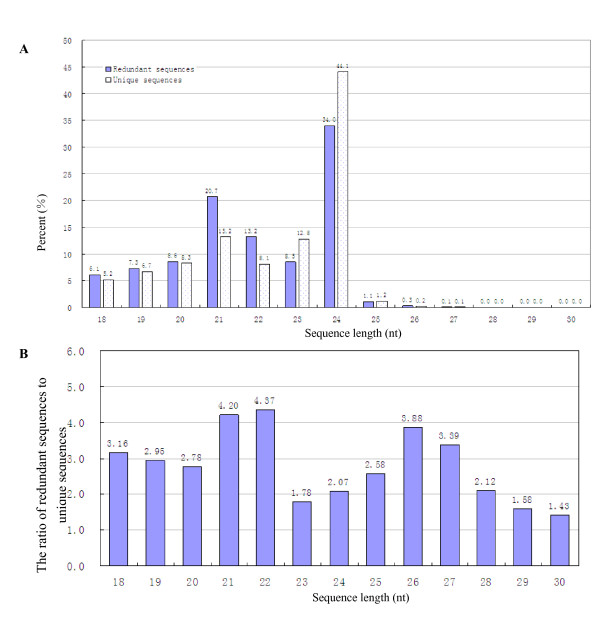
**Sequence length distribution of *C. trifoliata *sRNAs**.

**Figure 2:**
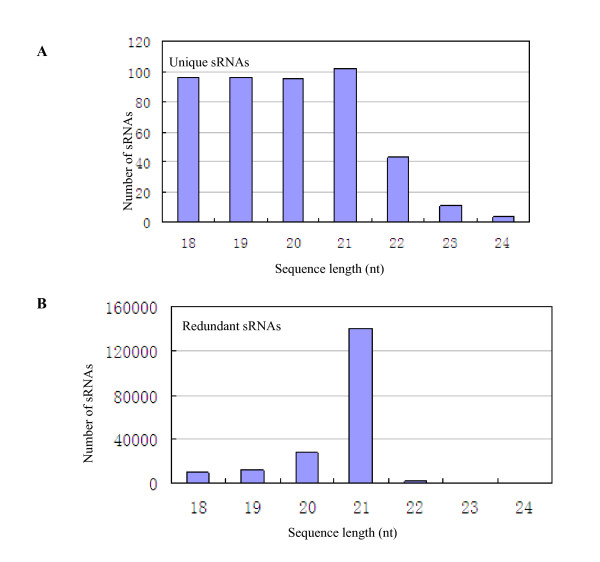
**Mapping of *C. trifoliata *sRNAs to other plant miRNAs**.

The size distribution of all sRNAs is as summarized in Figure 1A. The length of the trifoliate orange sRNAs varied from 18 nt to 30 nt, and the majority of them (approximately 92%) were in the range from 19 to 24 nt in length with 21 (13.2%) and 24 (44.1%) nt ones as the two major size classes (Figure 1A). This result was consistent with those of *Arabidopsis *[12], *Medicago truncatula *[24], and *Oryza sativa *[32]. In *Arabidopsis*, the 24 nt sRNAs even accounted to about 60% of its sRNA transcriptome [28]. However, the trifoliate orange sRNA size distribution differed from those of wheat and conifer obtained through 454 high throughput sequencing [20,33] and from *Taxus chinensis *ones obtained through Solexa sequencing [27]. To further compare the average abundance of sRNAs with different lengths, we measured the ratio of raw and unique sequences. SRNAs varied widely in length, and there is variation in redundancies of them, among which the sRNAs class with 21 and 22 nt showed the highest redundancies (Figure 1B). The average ratio of redundant and unique sequences of sRNAs with different sizes showed no obvious changes. Although the sRNAs annotated as miRNAs in the sizes of 18 nt, 19 nt, 20 nt, and 21 nt all had about 96-102 unique reads, their redundant reads surprisingly varied widely especially for the 21 nt group (Figure 2) which had 140,004 redundant reads and occupied almost 73% of the sRNAs assigned to miRNA therefore indicating that 21 nt long sRNAs are the most outstanding miRNA.

### Identifying conserved miRNAs in *C. trifoliata*

**Figure 3:**
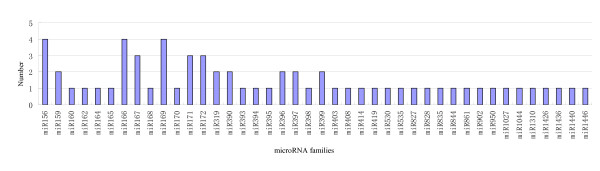
**Numbers of identical miRNA member in each family in *C. trifoliata***.

To identify the conserved miRNAs from the *C. trifoliata*, sRNA sequences identified from *C. trifoliata *by deep sequencing were compared with the currently known mature plant miRNAs in miRBase [34]. After Blastn searches and further sequence analysis, a total of 63 conserved miRNAs, belonging to 42 miRNA families, were identified in *C. trifoliate*. 23 miRNA* of these miRNAs were also sequenced (Additional file 1, Figure 3). The most of the identified miRNA families have been shown to be conserved in a variety of plant species using a comparative genomics-based strategy. For example, miR319, miR156/157, miR169, miR165/166, and miR394 have been found in 51, 45, 41, 40, and 40 plant species, respectively [9]. There is only one member identified in the majority of miRNA families, whereas some miRNA families contained many potential members (Figure 3) that need some further validation based on genomic or EST sequences.

It has been demonstrated that high throughput sequencing can provide an alternative way to estimate the expression profiles of miRNA genes [27,35] and allow us to determine the abundance of various miRNA families and even distinguish between different members of a given family of one organism. The highly expressed miRNA will likely have a large number of sequenced clones. Among the 42 miRNA families, the miR172 family had the most reads, accounting for 22.5% of the conserved miRNA reads. Additionally, fifteen miRNA families namely miR156, miR159, miR160, miR162, miR164, miR166, miR167, miR168, miR169, miR171, miR172, miR390, miR394, miR403, and miR1446, were found to have some thousands to tens of thousands of redundancies while four families (miR395, miR396, miR397, miR414, and miR827), had more than one hundred redundancies. The remaining families were infrequently sequenced (less than 100). In this study, we have tried to identify the precursor sequences for the 63 conserved *C. trifoliata *miRNAs, and only 29 pre-miRNAs and their secondary structures have been identified from the available citrus EST databases [9,29-31].

### Identifying novel potential miRNAs in *C. trifoliata*

**Figure 4:**
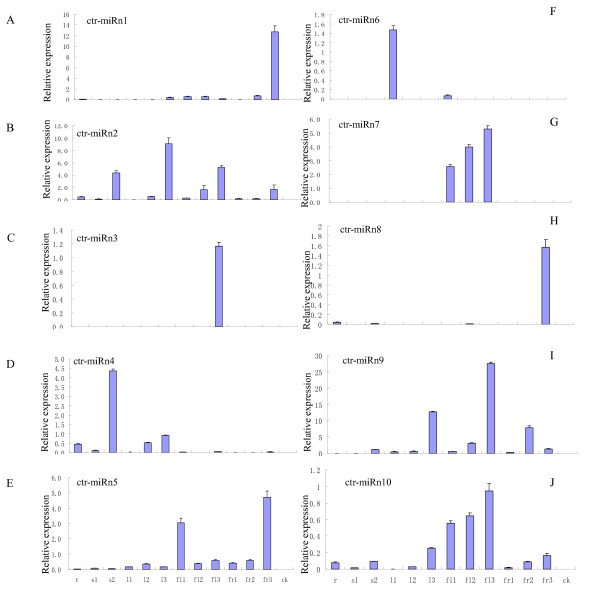
**Expression patterns of novel miRNA predicted from *C. trifoliata***. qRT-PCR of Low Molecular Weight RNA isolated from tissues at different development stages. *Lanes: *r, root; s1, young stem; s2, old stem; l1, leaf1 (0.2 cm in diameter); l2, leaf2 (0.5 cm in diameter); l3, leaf3 (1 cm in diameter); fl1, flower bud; fl2, partly open flower; fl3, full open flower; fr1, fruit 1 (about 0.5 cm in diameter); fr2, fruit 2 (about 1.5 cm in diameter); and fr3, fruit 3 (about 2 cm in diameter). Each reaction was repeated three times and the template amount was corrected by 5.8 s rRNAs.

Since the whole genomic sequence of *C. trifoliata *is unavailable, unique sRNA sequences were mapped to EST sequences restored in NCBI using *C. trifoliata*, as well as the other citrus species. By searching all *C. trifoliata *sRNAs against ESTs and predicting the secondary structures of a series of sequences surrounding them (Additional file 2), we identified 10 sequences that satisfied the secondary structure criteria as established by Zhang *et al. *[29] and shown in Table 3 with all the sequences meeting the new criteria of miRNA annotation [10]. Five of these putative miRNAs i.e. 50% were supported by miRNA*. Based on their near perfect secondary structure and following recent miRNA annotation criteria, these miRNAs were considered novel miRNAs [10]. Although not all were supported by miRNA*s, the novel *C. trifoliata*-specific miRNAs generally have a significant number of reads in the small library. Similar to conserved miRNAs, 8 of the 10 novel miRNAs begin with a 5' uridine, which is a characteristic feature of miRNAs (Table 3). The expression of the 10 miRNAs was assayed using qRT-PCR analysis and signals were detected for all of them (Figure 4). miRNAs specific to *C. trifoliata *exhibited different tissue-specific expression patterns. For example, Ctr-miRn1 was found to be specifically expressed in fruit tissue, whereas ctr-miRn2, which targeted *NB-LRR *gene (see below), was expressed in all the tissues assayed (Figure 4).

**Table 3:** Novel miRNA candidates in *C. trifoliata*

ID	**Sequence (5'**→**3')**	Precursor accession	A+U (%)	MFES	Location	LM	LP	No reads	RT-PCR	miRNA*
ctr-miRn1	AGAGAUCAAGUUGCAGAGCAA	EX447269	69.9	19.2	5'	21	83	41	+	Yes
ctr-miRn2	UUAAGAUUGAGUUACCAUCAU	EY823533	55.8	18	5'	21	86	5	+	Yes
ctr-miRn3	AUAAUGAUGUCUGUGAUGCCU	EY674266	55.4	21.7	3'	21	81	14	+	Yes
ctr-miRn4	UAGACCGCAAGAGACUAGCAA	EY845571	54.9	22.9	3'	21	82	37	+	Yes
ctr-miRn5	UGAAGGUCCGAGGUCGAGGUU	EY669005	66.3	19.8	3'	21	83	10	+	No
ctr-mi Rn6	UAAAUGUUGAGAGGAUUUGGC	DC890743	67.1	21	3'	21	76	5	+	No
ctr-miRn7	UGAGCGGCUGAAAAGAGGGAGAAA	DC893595	61.8	18.2	5'	24	89	6	+	No
ctr-miRn8	UAGGUGUAGAGAAGCACGAGA	CV712896	64.7	18.7	3'	21	85	10	+	No
ctr-miRn9	UUAGGGAUAUAACAGUUGAAU	CX297999	71.3	22.2	5'	21	73	110	+	No
ctr-miRn10	UUUCUUCAUGAGAGCUGGCCA	DT214737	47.1	54.5	5'	21	87	268	+	Yes

### Detection and expression patterns of novel potential miRNAs in *C. trifoliata*

Preferential expression of novel miRNAs in specific tissues might provide clues about the physiological function of these miRNAs. qRT-PCR is a reliable method for detecting and measuring the expression levels of miRNAs. In this study, we adopted this technique to validate and measure the expression of 10 novel potential miRNAs (ctr-miRn1, ctr-miRn2, ctr-miRn3, ctr-miRn4, ctr-miRn5, ctr-miRn6, ctr-miRn7, ctr-miRn8, ctr-miRn9 and ctr-miRn10). All of these potential miRNAs were identified in *C. trifoliata *by Solexa sequencing. To aid in determination of *C. trifoliata *novel miRNA functions, we examined their expression in different organs (Figure 4) by qRT-PCR analysis of LMW-RNA samples from various tissues of trifoliate orange trees. The data obtained can form powerful evidence to support the existence of the novel miRNAs in *C. trifoliata*. The expression patterns of most novel miRNAs in *C. trifoliata *appear to be tissue or development stage specific, with all the expression patterns of these miRNAs in citrus being grouped into several situations. The expression patterns of ctr-miRn1 and ctr-miRn8 were similar and revealed to be specifically expressed in *C. trifoliata *fruit tissue (fruit diameter = 2 cm) as shown in Figure 4A and 4H. However, ctr-miRn2 displayed different expression patterns in various organs and development stages where there was high expression in leaves of 1 cm diameter, weaker expression in leaves of 0.5 cm diameter and no expression in leaves of 0.2 cm diameter. Furthermore, ctr-miRn2 had different expression patterns in stem, flower, and fruits. It seems that ctr-miRn2 becomes strongly expressed as different tissues age. Some of the miRNAs might display species-specific and/or developmental stage-specific expression patterns, as exemplified by ctr-miRn3, ctr-miRn6 and ctr-miRn7 (Figure 4C, F and 4G). Ctr-miRn3 had preferential expression only in open flowers, while ctr-miRn6 had strong expression in leaves of 0.2 cm in diameter and weak expression in developing flower bud. Ctr-miRn7 was expressed only in flowers at different development stages (Figure 4H), while ctr-miRn4 seemed to be expressed strongly in old stems, moderately in leaves of 0.2 cm in diameter and weakly in roots of trifoliate orange (Figure 4D). Ctr-miRn5 was expressed in all tissues tested with the highest expression level in fruits of 2 cm in diameter and relatively strong expression in flower buds (Figure 4E). Ctr-miRn9 was expressed in all tissues tested except in root and young stem, with at highest expression level in flower and relatively strong expression in leaves of 1 cm in diameter (Figure 4I). Ctr-miRn10 was expressed in most of tissues except in leaves of 0.2 cm diameter. Specifically, ctr-miRn10 had strong expression in flowers at different stages and moderate expression in fruits of different sizes (Figure 4J).

In summary, 10 novel potential miRNAs, all identified by Solexa sequencing, were validated by qRT-PCR with some being expressed ubiquitously in all tissues with tissue, species, and/or growth stage specific characteristics reflected at different expression levels.

### Prediction of the *C. trifoliata *miRNA target genes

To better understand the functions of the newly identified species-specific as well as conserved *C. trifoliata *miRNAs, putative targets of these miRNAs were predicted using the described criteria and methods. We predicted target genes, and putative targets were identified for 24 out of 42 conserved families (Additional file 1). We also found homologs of known miRNA target genes for several conserved *C. trifoliata *miRNAs, such as SBP for miR156, ATP synthase for miR159, ARF for miR160, NAC for miR164, HD-Zip for miR165 and miR166, Anthocyanidin synthase for miR169, GRAS for miR171, AP2 for miR172, TCP for miR319, TIR for miR393, F-box for miR394, Sulfate transporter 2.1 for miR395, IRX12 copper ion binding/oxidoreductase for miR397, ARGONAUTE 2 for miR403, Basic blue copper protein for miR408 and Zinc finger protein-related for miR414. Additionally, we predicted a few genes with unknown function and hypothetical genes for miRNA targeting (Additional file 1). Careful analysis of these potential targets will contribute to our understanding of the role of miRNAs in fruit trees. No targets were found in *C. trifoliata *for miR398, miR399, miR419, miR530, miR535, miR827, miR828, miR35, miR844, miR861, miR902, miR950, miR1027, miR1044, miR1310, miR1426, miR1436, and miR1440.

Using the criteria used by Song et al. [30], targets were predicted for 3 of these novel miRNAs. It has been confirmed that miR172 targets the mRNA coding for *APETALA2*-like transcription factors, an important gene known for controlling flower development [36-38]. The important targets included *NB-LRR *disease resistance gene analogs, such as UC46-24238 and UC46-17900 that were found to be similar to *Populus trichocarpa *XM_002328188 cc-nbs-lrr resistance protein and UC46-20080 that was similar to *P. trichocarpa *XM_002301537 cc-nbs-lrr resistance protein.

### Identification of miRNA-guided cleavage of target mRNAs in *C. trifoliata*

**Figure 5:**
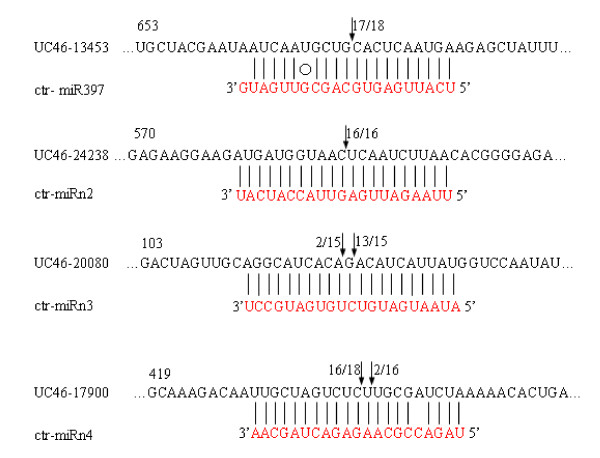
**Mapping of mRNA cleavage sites by RNA ligase-mediated 5' RACE**. Each top strand (black) depicts a miRNA-complementary site in the target mRNA, and each bottom strand (red) depicts the miRNA. Watson-Crick pairing (vertical dashes) and G:U wobble pairing (circles) are indicated. Arrows indicate the 5' termini of mRNA fragments isolated from Citrus, as identified by cloned 5'RACE products, with the frequency of clones shown. Only cloned sequences that matched the correct gene and had 5' ends within a 100 nt window centered on the miRNA complementary site are counted. Partial mRNA sequences from target genes were aligned with miRNAs. Numbers indicate the fraction of cloned PCR products terminating at different positions. UC46-13453 was similar to AT2G38080 (NM_129364) IRX12 (IRREGULAR XYLEM 12); laccase (IRX12); UC46-24238 and UC46-17900 were similar to *Populus trichocarpa *(XM_002328188) cc-nbs-lrr resistance protein; UC46-20080 was similar to *Populus trichocarpa *(XM_002301537) cc-nbs-lrr resistance protein.

Most *Arabidopsis *miRNAs have been shown to guide cleavage of their target genes [1,39]. To verify the nature of the potential miRNA targets and to study how the miRNAs in *C. trifoliata *regulate their target genes, a modified RLM-RACE experiment was set up, as described in the materials and methods section. In this study, the RLM-RACE procedure was successfully used to map the cleavage sites in four predicted target genes of *C. trifoliata*. Given the clear tissue-specific pattern of expression of ctr-miR397, ctr-miRn1, ctr-miRn2 and ctr-miRn3 in all types of vegetative organs of trifoliate orange (Figure 4), these analyses were performed on a few of their putative targets, using RNA extracted from leaves, stems, roots, flowers, and fruits of trifoliate orange, where ctr-miR397, ctr-miRn2, ctr-miRn3 and ctr-miRn4 were all abundantly expressed. UC46-13453, UC46-20080, UC46-17900, and UC46-24238 were confirmed as the real targets of ctr-miR397, ctr-miRn2, ctr-miRn3 and ctr-miRn4 respectively, since all the 5'ends of the mRNA fragments were mapped to the nucleotide that pairs to the tenth nucleotide of each miRNA with higher frequencies than depicted for each pairing oligo (Figure 5). All four predicted targets were found to have specific cleavage sites corresponding to the miRNA complementary sequences (Figure 5) and might be regulated by the four miRNAs in the style of small interfering RNAs (siRNAs) directing the cleavage of mRNA targets [40,41]. UC46-13453 is similar to *Arabidopsis *proteins coded by IRX12 copper ion binding/oxidoreductase (IRX12CBO) (Table 1), while UC46-20080, UC46-17900 and UC46-24238 all coded for a protein highly homologous to NB-LRR disease resistance protein (Table 4).

**Table 4:** Predicted targets for identified novel miRNAs in *C. trifoliata*.

ID	miRNA(3'-5')/mRNA(5'-3')	Target unigene no. (number of mismatches)	Target protein	Target function	Conserved gene in other plants (E-score)
ctr-miRn1	3'AACGAGACGUUGAACUAGAGA5'	No			
ctr-miRn2 (R)	3'UACUACCAUUGAGUUAGAAUU5'				
	AUGAUGGUAACUCAAUCUUAA	24238 (0)	disease resistance protein (NBS-LRR class)	defense response	*Populus trichocarpa *(2e-58)
ctr-miRn3 (R)	3'UCCGUAGUGUCUGUAGUAAUA5'				
	AGGCAUCACAGACAUCAUUAU	20080 (0)	disease resistance protein (NBS-LRR class)		*Populus trichocarpa *(5e-007)
ctr-miRn4 (R)	3'AACGAUCAGAGAACGCCAGAU5'				
	UUGCUAGUCUCUUGCGAUCUA	17900 (1)	disease resistance protein (NBS-LRR class)	defense response	AT3G14470 (2e-56)
ctr-miRn5	3'UUGGAGCUGGAGCCUGGAAGU5'	No			
ctr-miRn6	3'UAAAUGUUGAGAGGAUUUGGC5'	No			
ctr-miRn7	3'AAAGAGGGAGAAAAGUCGGCGAGU5'	No			
ctr-miRn8	3'AGAGCACGAAGAGAUGUGGAU5'	No			
ctr-miRn9	3'UAAGUUGACAAUAUAGGGAUU5'	No			
ctr-miRn10	3'ACCGGUCGAGAGUACUUCUUU5'				
	UGGCCAGCUCUCAUGAAGAAA	42150 (0)			

R, 5' RACE of target mRNA was done

## Discussion

### *C. trifoliata *conserved miRNAs

Although many miRNAs had been identified by traditional Sanger sequencing or computational approaches [30,42], most species-specific miRNAs are still unidentified. This is because traditional sequencing methods tend to generate a relatively small number of sequences while the plant species-specific miRNAs are often present at a lower level than the conserved miRNAs [11,12]. Thousands of plant miRNA genes have been annotated and some of them have been well characterized [16]. However, most plant miRNAs appears to be the conserved ones and many other functional miRNAs in plant species remain to be investigated. Compared to annotated miRNAs from *Arabidopsis *and rice, much fewer miRNAs from *C. trifoliata *plants have been identified. Recently, several studies performed in silico identification of miRNAs from citrus [9,29-31] and about 29 conserved miRNA families were detected, which is still not of a large number of the citrus miRNAs. Pursuing new sequencing technology can be good choice for the discovery of more miRNAs in citrus. Solexa deep sequencing platform is an ideal choice for miRNA discovery, and the sequencing results in this study showed its capability in that no complete genome is available. As to the miRNAs computationally predicted, most of them could also be discovered by this deep sequencing, except two of them (e.g. miR482 and miR435) [9,29-31]. It might be due to the spatiotemporal expression and depth of sequencing that miR482 and miR435 could not be sequenced. Deep sequencing can provide us the precise sequences of the miRNAs in an organism. The miRNAs sequenced in this study can definitely provide the information of *C. trifoliate *miRNAs for further study on their gene regulation function, the evolution and biogenesis of them.

### Novel miRNAs in *C. trifoliata*

Besides the conserved miRNAs, we also report 10 novel miRNAs by deep sequencing. Although the *C. trifoliata *full genome sequence is not available, the large number of citrus expressed sequence tags is an excellent source for precursor identification. Based on BLASTn search and hairpin structure prediction, we found potential precursors for all the 10 novel miRNAs. Because these 10 miRNAs were not similar to any known conserved miRNAs, and were predicted from citrus ESTs, then they might be specific to citrus and could play more species-specific roles. Prediction of these novel miRNAs involves the identification of stem-loop structure of the potential pre-miRNAs and sequencing of the miRNA* molecules [10]. All the 10 novel non conserved potential miRNAs more, 5 of these miRNA* yielded more than one read. In this study, qRT-PCR was found to be a reliable method for detecting and measuring the expression levels of miRNAs. We adopted this technique to validate and measure the expression of 10 novel miRNAs in trifoliate orange tissues (root, young and old stems, leaves of different sizes, flower bud, flower, and fruits at different development stages). All of these novel miRNAs were identified in trifoliate orange by Solexa sequencing. The qRT-PCR results demonstrate that most tested novel miRNAs were expressed with tissue-, species-, and/or growth-stage-specific characteristics, while a few were expressed ubiquitously in all tissues. Moreover our qRT-PCR results can not only validate the miRNA prediction in *C. trifoliata*, but also show that their preferential expression can provide important clues about where these miRNAs function. However, more studies need to be performed to elucidate the functions that these novel miRNAS have in the growth and development of trifoliate orange.

### miRNA target prediction

To assess and define a putative function for a miRNA in plant, a further step of target identification is necessary. Currently, the most efficient tool available for this is the bioinformatics approach facilitated by the high degree of homology between miRNA and its target sequences in plants [44]. Analysis of several targets has now confirmed this prediction, making it feasible to identify plant miRNA targets [1]. We first searched candidate targets of the citrus miRNAs and the putative miRNAs by Blast, and confirmed them by alignment with their orthologs in *Arabidopsis*. Our analysis reveals that most of the predicted targets in citrus have a conserved function with miRNA targets in *Arabidopsis *and these miRNA target sequences are also highly conserved among a wide variety of plant species as reported by Floyd and Bowman [45]. Consistent with previous reports, most of these targets in citrus were plant-specific transcription factors, such as *AP2*, *NAC*, *SBP *and the *ARF *family.

A large number of plant miRNA targets predicted bioinformatically have also been experimentally confirmed. Even though miRNAs generally function as negative regulators of gene expression by mediating the cleavage of target mRNAs [1] or by repressing their translation [2], the cleavage of target mRNAs appears to be the predominant mode of gene regulation by plant miRNAs [46]. Finding the cleavage site supposedly located in the sequence complementary to miRNA in the target gene is necessary to verify the cleavage of target mRNAs. Among the methods used to observe miRNA-dependent cleavage of targets, RLM-RACE is the most useful [1]. We performed the RACE on unigene to detect and clone the mRNA fragment corresponding precisely to the predicted product of miRNA processing. In total, we performed 5' RACE assays on four predicted target genes i.e. the representative targets of four conserved miRNAs (UC46-13453, UC46-20080, UC46-17900, and UC46-24238 were confirmed as the real targets of ctr-miR397, ctr-miRn2, ctr-miRn3 and ctr-miRn4 respectively). UC46-13453 is similar to *Arabidopsis *proteins coded by IRX12 copper ion binding/oxidoreductase (IRX12CBO) (Table 1), while UC46-20080, UC46-17900, and UC46-24238 all coded for a protein highly homologous to NB-LRR disease resistance protein. Targeting of NB-LRR genes by miRNAs has previously been reported in poplar [28], *Arabidopsis *[15], loblolly pine [47], and grape [13], but its contribution to disease resistance is still poorly characterized. In our study, all four predicted targets were found to have specific cleavage sites corresponding to the miRNA complementary sequences (Figure 5), and the most common 5' end of the mRNA fragments mapped to the nucleotides that pair with the 10^th ^miRNA nucleotide from the 5' ends. This validation was obtained by performing the modified 5' RACE protocol on mRNA extracted from pooled tissues of leaf, stem, root, and flower where it had been previously demonstrated that ctr-miRn2, ctr-miRn3, and ctr-miRn4 are all abundant (Figure 4B, C, and 4D). miRNAs may directly target transcription factors that affect plant development and also specific genes that control metabolism. In our study, it appears that our predicted targets play roles not only in development, but also in diverse physiological processes.

## Conclusion

For the first time we discovered through high through-put Solexa sequencing of short RNAs from *C. trifoliata *flowers and fruits 10 new potential miRNAs and 42 highly conserved miRNA families, indicating that specific miRNAs exist in citrus species. These results show that regulatory miRNAs exist in agronomically important trifoliate orange and may play an important role in citrus growth, development, and response to disease.

## Methods

### Plant material

Flower buds, partly-open and fully-open flowers, and developing fruits in different sizes (0.3, 0.5, 0.8, 1, and 1.5 cm in diameter) were collected from five-year old trifoliate orange (*Citrus trifoliata*.) trees at the Tree Fruit Research and Extension Center, Suzhou Fruit Tree Research Institute, China in 2009. After collection, all the samples were immediately frozen in liquid Nitrogen and stored at -80ºC until used.

### RNA isolation

Following the manufacturer's instructions, total RNA was isolated from the above described tissues by using Trizol (Invitrogen, Life Technologies, Carlsbad, CA). 100 mg of plant tissues were ground with liquid Nitrogen followed by thorough mixing with 2 mL lysis solution and transfer to a centrifuge tube. After placing on ice for 30 min, the mixtures were centrifuged for 15 min at 11,000 g and 4°C. The upper aqueous phase was transferred into a new centrifuge tube and 500 μL of extraction reagent was added and thoroughly mixed on ice for 15 min, the mixtures were then centrifuged for 15 min at 11,000 g and 4°C. The upper aqueous phase was further transferred into another centrifuge tube and 500 μL of isopropanol added to each sample. After precipitating for 30 min at 37°C, the mixtures were centrifuged for 15 min 12,000 g and 4°C. The supernatant was discarded and the RNA pellet was dried. The RNA pellet was then washed using 1 mL 75% ethanol and total RNA precipitated by centrifuging for 5 min 7,500 g and 4°C. After this, RNA pellets were completely dried, then dissolved in 50 μL of RNase free water, and stored at -80°C until sRNA sequencing. All the RNA samples from different tissues were mixed to form a single RNA pool.

### SRNA sequencing and sequence processing

SRNA samples were sequenced by Beijing Genomics Institute (BGI) (Shenzhen, Guangdong, China) using the high throughput pyrosequencing technology developed by Illumina. SRNAs with 16-30 nt in length were first separated from the total RNA by size fractionation with 15% TBE urea polyacrylamide gel (TBU). The sRNA (16-30 nt in length) were excised from the gel and submerged in 600 μL of 0.3 M sodium chloride overnight at 4°C. The gel slurry was then passed through a Spinfilter column (Corning, Beijing, China), and RNA precipitated by addition of 3 μL of 5 mg/mL mussel glycogen (Invitrogen, Carlsbad, CA, USA) and 800 μL of ethanol. The RNA pellets were then washed with 75% ethanol and air dried at 25°C. The sRNA was resuspended in 5.0 μL of diethylpyrocarbonate (DEPC) treated water (Ambion, Austin, TX, USA). The isolated sRNAs were then ligated to 5' adaptor (5'UCAGAGUUCUACAGUCCGACGAUC) using T4 RNA ligase (Promega, Madison, WI) in the presence of RNase Out (Invitrogen) overnight at 4°C according to the manufacturer's instructions. After selecting the ligated products by size fractionation, a 3'adaptor (5'UCGUAUGCCGUCUUCUGCUUGUidT) was ligated to the sRNAs following the same procedure as the ligation of the 5'adaptor. Finally, the ligated RNAs were size fractionated on a 10% TBE urea polyacrylamide gel and the 70 nt RNAs were excised. The 3'adaptorgated sRNAs were then purified from the gel and precipitated as described above followed by re-suspension in 5.0 μL DEPC treated water (Ambion). The sRNA with 5' and 3'adaptors were selected and reversely transcribed to cDNA with the RT primer (CAAGCAGAAGACGGCATACGA) using Superscript II reverse transcriptase (Invitrogen). The cDNA was further purified by 15% TBU followed by dissolving in 100 μL1 × NEB. The cDNA was quantified by Agilent 2100 and diluted to 10 nM at final concentration. 18 ng cDNA was loaded into the Illumina 1 G Genome Analyzer for sequencing.

### Bioinformatic analysis of miRNA identified

The raw sequences were processed as described by Sunkar et al. [16,46]. After removing the vector sequences, modified sequences from 18 nt to 30 nt were used for further analyses. First, rRNA, tRNA, snRNA, and snoRNA, as well as those containing the polyA tail, were removed from the sRNA sequences and the remaining sequences were compared against rice and *Arabidopsis *ncRNAs deposited in the NCBI Genbank database and Rfam8.0 database. Then, the unique sRNA sequences were used to do a Blastn search against the miRNA database [34], miRBase 13.0, to identify the conserved miRNAs in *C. trifoliata*. Only the perfectly matched sequences were considered to be conserved miRNAs.

To study potential miRNA precursor sequences, all 560, 271 citrus ESTs were downloaded from the National Center for Biotechnology Information (NCBI) GenBank EST database (March 2009; http://www.ncbi.nlm.nih.gov/, including data from *Citrus sinensis *(207,500), *C. clementina *(118,365), *C. trifoliata *(58,483), *C. reticulata *(55,319) and all other taxa (120,604). The ESTs were aligned to all the sRNA sequences from *C. trifoliata*, and then the miRNA candidates were processed by miRCat http://srna-tools.cmp.uea.ac.uk/; [43]), using default parameters, to generate the secondary structures.

### Prediction of potential target mRNAs for *C. trifoliata *miRNAs

Putative *C. trifoliata *miRNAs were first blasted against Harvest C46 Citrus unigene database on the Harvest Blast Search web server http://138.23.191.145/blast/index.html. BLASTn hits with less than four nucleotide mismatches (plus/minus) were chosen as the candidate targets, and were then searched in Citrus Harvest 1.20 program using BLASTx to obtain their putative functions. Meanwhile, citrus miRNAs were blasted with the citrus ESTs by Blast2.17 (Elue set as 10). ESTs with less than four nucleotide mismatches (plus/minus) were extracted and then annotated by the online BLASTx search against the Swissprot protein sequences (swissprot) or nondundant protein sequence (nr) database on the NCBI web server.

### Real-Time PCR of miRNAs

Roots, leaves (0.2, 0.5 and 1 cm in diameter), young stems, old stems, flower bud, half open flower, fully open flower, and fruits (0.5, 1.5, and 2 cm diameter) were collected from the trifoliate orange (the same as used for deep sequencing), and the total RNA was isolated from 100 mg of these tissues using TRIZOL reagent (Invitrogen, Life Technologies, Carlsbad, CA). Low molecular weight RNA and high molecular weight RNA were separated with 4 M LiCl [30,48]. SRNAs were polyadenylated at 37°C for 60 min in a 50 μl reaction mixture with 1.5 μg of total RNA, 1 mM ATP, 2.5 mM MgCl_2_, and 4 U poly(A) polymerase (Ambion, Austin, TX). Poly (A)-tailed sRNA was recovered by phenol/chloroform extraction and ethanol precipitation. The sRNAs were dissolved, treated with RNase-free DnaseI (Takara, Japan) and reversely transcribed using poly (T) adapter [49]. qPCR was performed using SYBR^® ^Green Realtime PCR Master Mix (Toyobo, Osaka, Japan) and all the primers used were as listed in Additional file 3. The values of threshold cycle (CT, the fractional cycle number at which the fluorescence passes the fixed threshold), were calculated by Rotor-Gene 6 software (Corbett Robotics, Australia). For each reaction, 1 μL of diluted cDNA (equivalent to 100 pg of total RNA) was mixed with 10 μL of 2× SYBR green reaction mix (SYBR^® ^Green qRT-PCR Master Mix; Toyobo, Osaka, Japan), and 5 pmol of the forward and the reverse primers were added to make a final volume 20 μL. The conditions for the PCR amplification were as follows: polymerase activation at 95°C for 1 min; followed by 50 cycles of 95°C for 15 s, 95°C for 15 s, 60°C for 20 s, and 72°C for 20 s. The fluorescence signal was measured once every 1°C. Negative PCR controls (no cDNA template) were prepared to detect possible contamination. The specificity of the primer amplicons was texted by analysis of a melting curve. The CT values were converted into relative copy numbers using a standard curve [50]. The 5.8S rRNA was used as a reference gene in the qPCR detection of miRNAs in *Arabidopsis *[51]. The data was analyzed with an R^2 ^above 0.998 using the LinRegPCR program [52].

### Identification of miRNA-mediated cleavage of target mRNAs in *C. trifoliata*

For mapping internal cleavage sites in the mRNA of UC46-13453, UC46-20080, UC46-17900, and UC46-24238 unigenes, which are targeted by ctr-miR397, ctr-miRn2, ctr-miRn3, and ctr-miRn4, respectively, RNA ligase-mediated rapid amplification of cDNA ends (RLM-RACE) was performed using a GeneRacer Kit (Invitrogen Life Technologies). Total RNA was extracted from the root, stem, leaf, flower and fruit tissues of an adult *C. trifoliata *tree using TRIZOL reagent then equivalent total RNA of different tissues were mixed. Poly(A)^+ ^mRNA was purified from all tissues using a PolyA kit (Promega, Madison, WI) according to manufacturer's instructions. A modified procedure for 5'-RLM-RACE was followed using the GeneRacer Kit as described previously [1,30]. A GeneRacer RNA Oligo adapter (5'CGACUGGAGCACGAGGACACUGACAUGGACUGAAGGAGUAGAAA 3') was directly ligated to mRNA (250 ng) without calf intestinal phosphatase and tobacco acid pyrophosphatase treatment. The GeneRacer Oligod_T _primer (5'GCTGTCAACGATACG CTACGTAACGGCATGACAGTG (T)_30 _3') was then used to synthesize the first-strand cDNA by reverse transcription. The cDNA was amplified by GeneRacer 5' primer and GeneRacer 3' primer to generate a pool of non-gene-specific 5'-RACE products. The conditions for this amplification were the same as those for gene-specific RACE recommended by the manufacturer, with the exception that an extension time of 3 min was employed. Gene-specific 5'-RACE reactions were performed using GeneRacer 5' nested primer and the following gene-specific primers: UC46-13453-832R (5'-GACATTGGTGGTTTGGCCTGGAGCTATTAC-3'), UC46-24238-648R (5'-CATGCTGAGT GGGACGGTATATTGG-3'), UC46-20080-492R (5'-GTGAAAGCGGCAGTTCTTCTAATG-3') and UC46-17900-786R (5'-TGGGCAAAGTCGTGCACTATATCAT-3'). In each case, a unique gene-specific DNA fragment was amplified. These products were gel purified and cloned. At least 15 independent clones from each reaction were sequenced.

### Data access

The sRNA sequence data from this study have been submitted to Gene Expression Omnibus (GEO) under accession No. GSE22089 at website: http://www.ncbi.nlm.nih.gov/geo/query/acc.cgi?token=jjqnhwgaagisqfm&acc=GSE22089.

## Authors' contributions

SC carried out the laboratory work and participated in manuscript draft writing. ZC performed bioinformatics analyses. YH and MZ participated in the design and coordination the study. WC constructed the sRNA library. KKN revised this paper. FJ conceived, designed the study and revised this paper. All authors read and approved the final manuscript.

## Supplementary Material

Additional file 1 **Conserved miRNAs in C. trifoliata sRNA library**. Click here for file

Additional file 2 **Secondary structures of novel potential miRNAs in *C. trifoliata***. Red colored letter: mature miRNA sequence; pick colored letter: miRNA* sequence. Click here for file

Additional file 3 **The sequences of primer used for qRT-PCR validation of the novel miRNAs**. Click here for file
