# Supplementary material for: Deep sequencing discovery of novel and conserved microRNAs in trifoliate orange (Citrus trifoliata)
Source: BMC Genomics. 2010 Jul 13;11:431. doi: 10.1186/1471-2164-11-431 (PMC2996959; doi:10.1186/1471-2164-11-431)
Supplement: Additional file 1 — Conserved miRNAs in C. trifoliata sRNA library. [file 1471-2164-11-431-S1.DOC]

**Additional file 1. Conserved miRNAs in *C. trifoliata* sRNA library**

| miRNA family | Name | sequence (5’→3’) | Reads | precursor | MiRNA* sequenced | Homolog | Predicted target family |
| --- | --- | --- | --- | --- | --- | --- | --- |
| 156 | ctr-miR156a | TGACAGAAGAGAGTGAGCAC | 15273 | Y | N | ath-miR156a | Squamosa-promoter binding protein |
|  | ctr-miR156b | CTGACAGAAGAGAGTGAGCAC | 5815 | N | N | smo-miR156b |  |
|  | ctr-miR156c | TTGACAGAAGAGAGTGAGCAC | 2544 | N | N | smo-miR156c |  |
|  | ctr-miR156d | TGACAGAAGAGAGTGAGCACA | 677 | N | N | bna-miR156a |  |
| 159 | ctr-miR159a | TTTGGATTGAAGGGAGCTCTA | 3852 | N | Y | ath-miR159a | ATP synthase |
|  | ctr-miR159b | TTTGGATTGAAGGGAGCTCTT | 24 | N | N | ath-miR159b |  |
| 160 | ctr-miR160a | TGGCGTATGAGGAGCCATGCA | 595 | Y | Y | ath-miR160a | Auxin response factor |
| 162 | ctr-miR162a | TCGATAAACCTCTGCATCCAG | 1691 | Y | Y | ath-miR162a | Zinc finger protein |
| 164 | ctr-miR164a | TGGAGAAGCAGGGCACGTGCA | 29536 | Y | Y | ath-miR164a | NAC domain protein |
| 165 | ctr-miR165a | TCGGACCAGGCTTCATCCCCC | 17 | Y | Y | ath-miR165a | HD-Zip protein |
| 166 | ctr-miR166a | TCGGACCAGGCTTCATTCCCC | 3026 | Y | Y | ath-miR166a | HD-Zip protein |
|  | ctr-miR166h | TCGGACCAGGCTTCATTCCC | 1320 | N | N | zma-miR166h |  |
|  | ctr-miR166j | TTCGGACCAGGCTTCATTCCC | 118 | N | N | ppt-miR166j |  |
|  | ctr-miR166d | TCGGACCAGGCTTCATTCCCCT | 5 | N | N | vvi-miR166d |  |
| 167 | ctr-miR167a | TGAAGCTGCCAGCATGATCTA | 10517 | Y | Y | ath-miR167a | Auxin response factor |
|  | ctr-miR167d | TGAAGCTGCCAGCATGATCTGG | 1570 | N | N | ath-miR167d |  |
|  | ctr-miR167e | TGAAGCTGCCAGCATGATCTG | 1014 | N | N | osa-miR167d |  |
| 168 | ctr-miR168a | TCGCTTGGTGCAGGTCGGGAA | 19065 | Y | Y | ath-miR168a | unknown |
| 169 | ctr-miR169a | AGCCAAGGATGACTTGCCGGA | 20 | Y | Y | ath-miR169a | Anthocyanidin synthase |
|  | ctr-miR169b | CAGCCAAGGATGACTTGCCGG | 1095 | N | N | ath-miR169b |  |
|  | ctr-miR169e | TAGCCAAGGATGACTTGCCTGC | 17 | N | N | osa-miR169e |  |
|  | ctr-miR169h | TAGCCAAGGATGACTTGCCTG | 32 | N | N | ath-miR169h |  |
| 170 | ctr-miR170 | TGATTGAGCCGTGTCAATATC | 1 | Y | N | ath-miR170 | unknown |
| 171 | ctr-miR171a | TATTGGCCTGGTTCACTCAGA | 117 | Y | Y | ath-miR171a | GRAS transcription factor |
|  | ctr-miR171b | TTGAGCCGTGCCAATATCAC | 124 | N | N | zma-miR171b |  |
|  | ctr-miR171c | TGATTGAGCCGTGCCAATATC | 1410 | N | N | osa-miR171b |  |
| 172 | ctr-miR172a | AGAATCTTGATGATGCTGCAT | 37575 | Y | Y | ath-miR172a | AP2 related transcription factor |
|  | ctr-miR172c | AGAATCTTGATGATGCTGCAG | 28 | N | N | ath-miR172c |  |
|  | ctr-miR172e | GGAATCTTGATGATGCTGCAT | 48 | N | N | ath-miR172e |  |
| 319 | ctr-miR319a | TTGGACTGAAGGGAGCTCCC | 5 | Y | Y | mtr-miR319 | TCP transcription factor |
|  | ctr-miR319e | TTTGGACTGAAGGGAGCTCCT | 4 | N | N | vvi-miR319e |  |
| 390 | ctr-miR390a-3p | CGCTATCCATCCTGAGTTTCA | 242 | N | Y | gma-miR390a-3p | unknown |
|  | ctr-miR390a | AAGCTCAGGAGGGATAGCGCC | 2835 | Y | N | ath-miR390a |  |
| 393 | ctr-miR393 | TCCAAAGGGATCGCATTGATC | 4 | N | Y | osa-miR393 | Transport inhibitor response-like protein |
| 394 | ctr-miR394a | TTGGCATTCTGTCCACCTCC | 1296 | Y | Y | ath-miR394a | F-box protein |
| 395 | ctr-miR395a | CTGAAGTGTTTGGGGGAACTC | 93 | N | Y | ath-miR395a | Sulfate transporter 2.1 |
| 396 | ctr-miR396a | TTCCACAGCTTTCTTGAACTG | 303 | Y | Y | ath-miR396a | Hypothetical protein |
|  | ctr-miR396b | TTCCACAGCTTTCTTGAACTT | 529 | N | Y | ath-miR396b |  |
| 397 | ctr-miR397a | TCATTGAGTGCAGCGTTGATG | 152 | N | Y | ath-miR397a | IRX12 copper ion binding/ oxidoreductase |
|  | ctr-miR397b | TCATTGAGTGCAGCGTTGATGT | 8 | N | N | bna-miR397a |  |
| 398 | ctr-miR398 | TTGTGTTCTCAGGTCACCCCT | 1 | Y | Y | ath-miR398 | - |
| 399 | ctr-miR399b | TGCCAAAGGAGATTTGCCCTG | 26 | Y | Y | ath-miR399b | - |
|  | ctr-miR399j | TGCCAAAGGAGAGTTGCCCTA | 61 | N | N | osa- miR399j |  |
| 403 | ctr-miR403 | TTAGATTCACGCACAAACTCG | 527 | Y | N | ath-miR403 | ARGONAUTE 2 |
| 408 | ctr-miR408 | ATGCACTGCCTCTTCCCTGGC | 8 | Y | Y | ath-miR408 | Basic blue copper protein |
| 414 | ctr-miR414 | TATCTTCTTCATCATCTTC | 111 | N | N | ath-miR414 | Zinc finger protein-related |
| 419 | ctr-miR419 | TGATGAATGATGAACGATGATGAC | 7 | Y | N | ppt-miR419 | - |
| 530 | ctr-miR530a | TGCATTTGCACCTGCACCTT | 4 | Y | N | ptc-miR530a | - |
| 535 | ctr-miR535a | TGACAATGAGAGAGAGCACACC | 1157 | N | N | ppt-miR535a | - |
| 827 | ctr-miR827 | TTAGATGACCATCAACAAACA | 114 | N | N | ath-miR827 | - |
| 828 | ctr-miR828 | TCTTGCTCAAATGAGTATTCCA | 4 | N | N | ath-miR828 | - |
| 835 | ctr-miR835-5p | TTTGTGTCATATGTTCTTTATC | 1 | Y | N | ath-miR835-5p | - |
| 844 | ctr-miR844 | TGGATGAGATTGCTTGTAACT | 1 | Y | N | ath-miR844 | - |
| 861 | ctr-miR861-5p | TGGTATATGGAGGAGGTAACTGAG | 18 | N | N | ath-miR861-5p | - |
| 902 | ctr-miR902-3p | AGAAGAATCTTGAAACATATA | 19 | N | N | ppt-miR902l-3p | - |
| 950 | ctr-miR950a | TCAGGTCTTGATGGTTTAT | 1 | Y | N | pta-miR950a | - |
| 1027 | ctr-miR1027 | TTTCTTATCTTCTTTTCATC | 40 | Y | N | ppt-miR1027a | - |
| 1044 | ctr-miR1044 | TTGAGGGCATATTTCTTTTA | 63 | Y | N | ppt-miR1044-3p | - |
| 1310 | ctr-miR1310 | AGGCATCGGGGGCGCAACGCCC | 10 | N | N | pta-miR1310 | - |
| 1426 | ctr-miR1426 | AGAATCTTGATGATGATGAAA | 22 | Y | N | osa-miR1426 | - |
| 1436 | ctr-miR1436 | ACATATATGGGACGGGAGTGAGA | 74 | N | N | osa-miR1436 | - |
| 1440 | ctr-miR1440 | TGCTCAAATACCACTCTCCT | 1 | N | N | osa-miR1440 | - |
| 1446 | ctr-miR1446 | TTCTAAACTCTCTCCCTCATA | 11772 | Y | N | ptc-miR1446a | GRAS family transcription factor |

Y, yes; N, no
