# Supplementary material for: Deep sequencing discovery of novel and conserved microRNAs in trifoliate orange (Citrus trifoliata)
Source: BMC Genomics. 2010 Jul 13;11:431. doi: 10.1186/1471-2164-11-431 (PMC2996959; doi:10.1186/1471-2164-11-431)
Supplement: Additional file 2 — Secondary structures of novel potential miRNAs in C. trifoliata. Red colored letter: mature miRNA sequence; pick colored letter: miRNA* sequence. [file 1471-2164-11-431-S2.DOC]

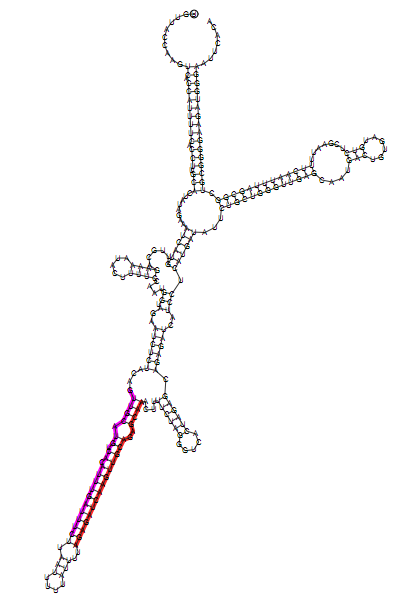


ctr-miRn1-5p: GUUGCAUGUCACAUUUGAUUUC

ctr-miRn1-3p: AGAGAUCAAGUUGCAGAGCAA


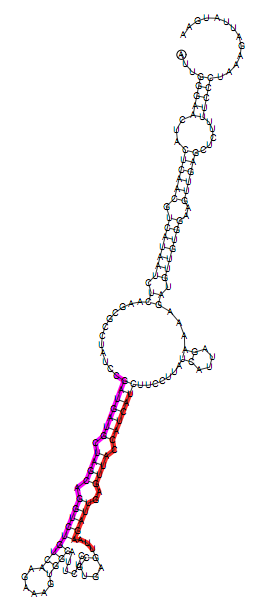


ctr-miRn2-5p: CGAUGAUGCUAGCAGGUCUGU

ctr-miRn2-3p: UUAAGAUUGAGUUACCAUCAU


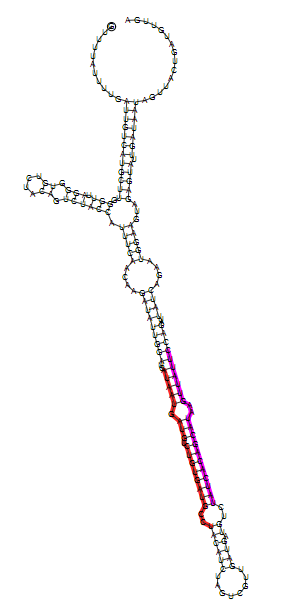


ctr-miRn3-5p: AUAAUGAUGUCUGUGAUGCCU

ctr-miRn3-3p: UAUCACAGCAUAAGUUAUUC


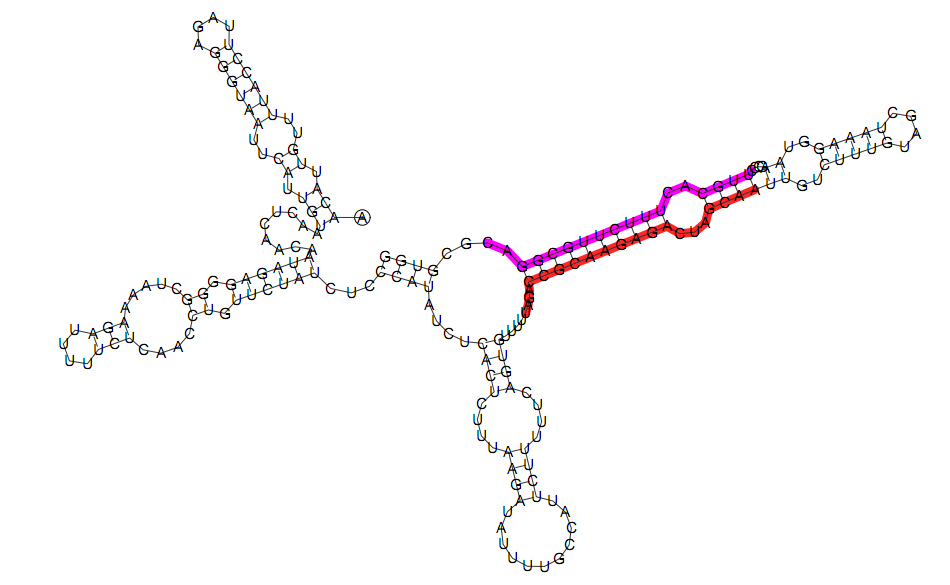


ctr-miRn4-5p: UAGACCGCAAGAGACUAGCAA

ctr-miRn4-3p: CUUGCACUUUCUUGCGGAC


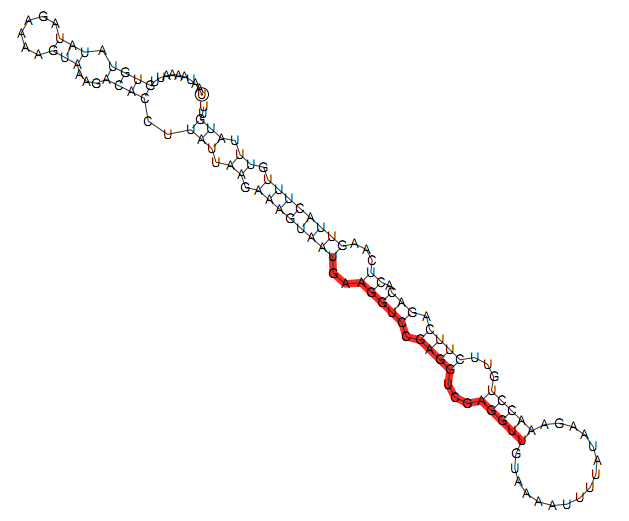


ctr-miRn5: UGAAGGUCCGAGGUCGAGGUU


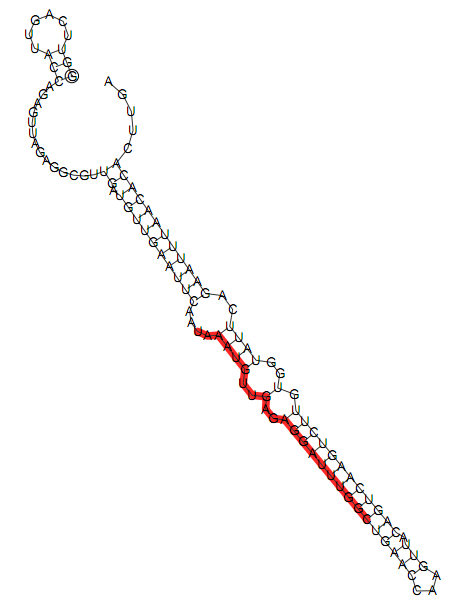


ctr-miRn6 : UAAAUGUUGAGAGGAUUUGGC


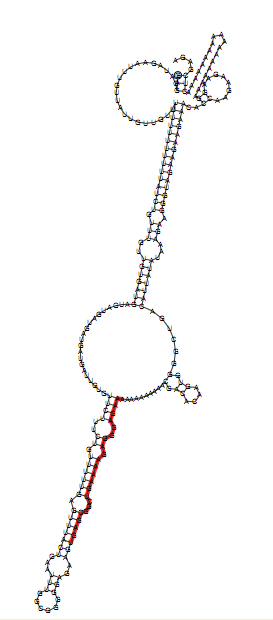


ctr-miRn7: UGAGCGGCUGAAAAGAGGGAGAA


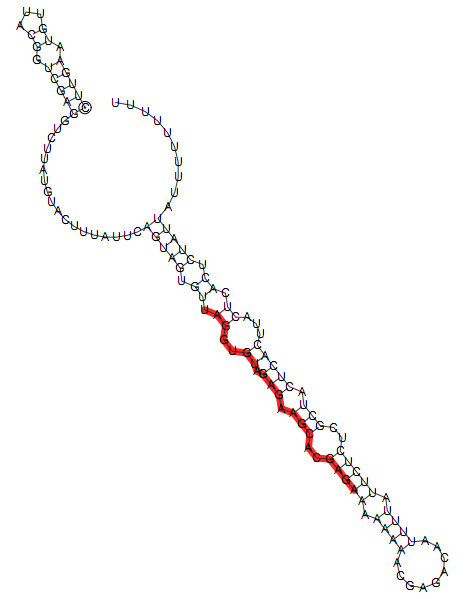


ctr-miRn8: UAGGUGUAGAGAAGCACGAGA


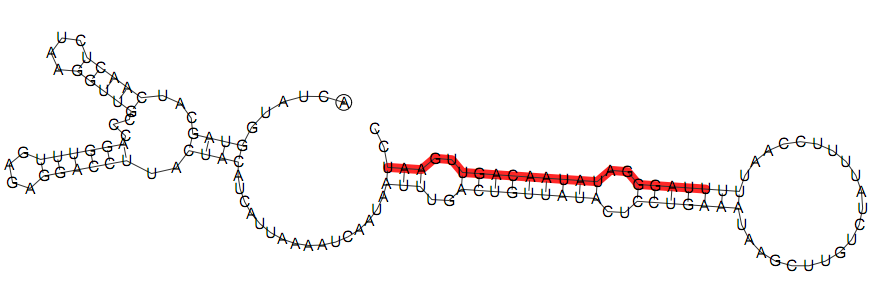


ctr-miRn9: UUAGGGAUAUAACAGUUGAAU


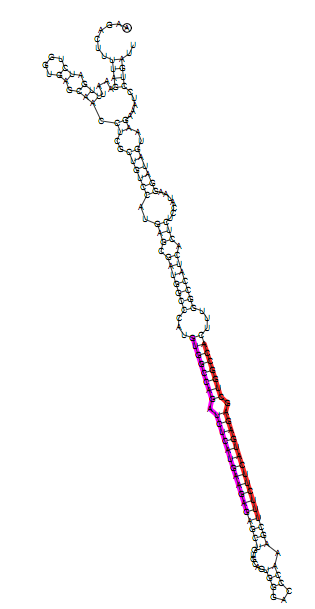


ctr-miRn10-5P: GUGGCCAGAUCUCAUGAAGAG

ctr-miRn10-3P: UUUCUUCAUGAGAGCUGGCCA
