# Supplementary material for: Deep sequencing discovery of novel and conserved microRNAs in trifoliate orange (Citrus trifoliata)
Source: BMC Genomics. 2010 Jul 13;11:431. doi: 10.1186/1471-2164-11-431 (PMC2996959; doi:10.1186/1471-2164-11-431)
Supplement: Additional file 3 — The sequences of primer used for qRT-PCR validation of the novel miRNAs. [file 1471-2164-11-431-S3.DOC]

**Additional file 3. qRT-PCR validated novel miRNAs and their sequences.**

| **miRNA** | **specific primers (5’ → 3’)** | **adaptor primers (5’ → 3’)** |
| --- | --- | --- |
| ctr-miRn1 | AGAGATCAAGTTGCAGAGCAA | ATTCTAGAGGCCGAGGCGGCCGACATG |
| ctr-miRn2 | TTAAGATTGAGTTACCATCAT | ATTCTAGAGGCCGAGGCGGCCGACATG |
| ctr-miRn3 | ATAATGATGTCTGTGATGCCT | ATTCTAGAGGCCGAGGCGGCCGACATG |
| ctr-miRn4 | TAGACCGCAAGAGACTAGCAA | ATTCTAGAGGCCGAGGCGGCCGACATG |
| ctr-miRn5 | TGAAGGTCCGAGGTCGAGGTT | ATTCTAGAGGCCGAGGCGGCCGACATG |
| ctr-mi Rn6 | TAAATGTTGAGAGGATTTGGC | ATTCTAGAGGCCGAGGCGGCCGACATG |
| ctr-miRn7 | TGAGCGGCTGAAAAGAGGGAGAAA | ATTCTAGAGGCCGAGGCGGCCGACATG |
| ctr-miRn8 | TAGGTGTAGAGAAGCACGAGA | ATTCTAGAGGCCGAGGCGGCCGACATG |
| ctr-miRn9 | TTAGGGATATAACAGTTGAAT | ATTCTAGAGGCCGAGGCGGCCGACATG |
| ctr-miRn10 | TTTCTTCATGAGAGCTGGCCA | ATTCTAGAGGCCGAGGCGGCCGACATG |
